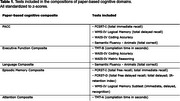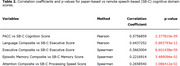# Associations between remote speech‐based and paper‐and‐pencil cognitive assessments in individuals with Subjective Cognitive Decline

**DOI:** 10.1002/alz70856_103437

**Published:** 2025-12-25

**Authors:** Clàudia Porta‐Mas, Gonzalo Sánchez‐Benavides, Elisa Mallick, Johannes Tröger, Nicklas Linz, Alexandra König, Andreea Rădoi, Carlota Medina Rodríguez, Alba Cañas‐Martínez, Anna Brugulat‐Serrat, Lidia Canals‐Gispert, Isabel Pérez‐Gutiérrez, Marc Suárez‐Calvet, Juan Domingo Gispert, Oriol Grau‐Rivera

**Affiliations:** ^1^ Barcelonaβeta Brain Research Center (BBRC), Pasqual Maragall Foundation, Barcelona, Spain; ^2^ Hospital del Mar Research Institute, Barcelona, Spain; ^3^ Centro de Investigación Biomédica en Red de Fragilidad y Envejecimiento Saludable (CIBERFES), Instituto de Salud Carlos III, Madrid, Spain; ^4^ ki:elements GmbH, Saarbrücken, Germany; ^5^ Hospital del Mar Research Institute, Barcelona, Barcelona, Spain; ^6^ Servei de Neurologia, Hospital del Mar, Barcelona, Spain

## Abstract

**Background:**

We aimed to provide validation data on Mili‐platform–a remote, automated speech‐based cognitive assessment conducted via telephone–by comparing speech‐derived cognitive scores with standard cognitive paper‐and‐pencil evaluations in a cohort of individuals with Subjective Cognitive Decline (SCD).

**Method:**

Data were collected from 91 participants (mean[SD]age:65.8[6.7]; 59,34% women; mean[SD]education:15.5[3.8]) with SCD, enrolled in the B‐AARC cohort in Spain, and participating in PROSPECT‐AD study, a multi‐cohort European longitudinal study aiming to develop algorithms to identify speech biomarkers for AD. The B‐AARC cohort included paper‐and‐pencil cognitive assessments, from which composite scores for executive function, episodic memory, and language were derived, as well as the PACC for a global cognition score. Speech samples were automatically collected during cognitive tasks via the Mili platform (ki:elements) by phone, including a semantic verbal fluency test and a four‐trial 15‐word Auditory Verbal Learning Test. These tasks generated speech‐based cognitive scores (SB‐C), measuring three cognitive domains—memory, processing speed, and executive function—and a global cognition score. Pearson and Spearman rank correlations were used to evaluate associations between SB‐C domain scores and paper‐and‐pencil cognitive domains.

**Result:**

SB‐C scores demonstrated moderate correlations with paper‐and‐pencil cognitive domains. The SB‐C global cognition score significantly correlated with the PACC (*r*:0.57; *p*‐value:<0.0001). The SB‐C executive function score correlated with the paper‐based executive function composite (*r*:0.56; *p*‐value:<0.0001), as well as with the paper‐based language composite (*r*:0.64; *p*‐value:<0.0001). The SB‐C memory score correlated with the paper‐based memory domain, though weakly (*r*:0.22; *p*‐value:0.03), as well as the SB‐C processing speed score and the paper‐based attention composite (*r*:0.26; *p*‐value:0.01).

**Conclusion:**

These findings provided validation on the utility of remote, automated speech‐based cognitive assessments in individuals at risk of AD compared to traditional assessments, offering a more scalable and accessible tool. Some domains displayed low correlations, suggesting that adding speech features to the composites provides additional information, potentially more sensitive and granular to actual changes, which may be not reflected in the paper‐pencil tests. Further studies on predicting brain pathology and cognitive decline are warranted.